# Metagenomic Analysis of Lysogeny in Tampa Bay: Implications for Prophage Gene Expression

**DOI:** 10.1371/journal.pone.0003263

**Published:** 2008-09-23

**Authors:** Lauren McDaniel, Mya Breitbart, Jennifer Mobberley, Amy Long, Matthew Haynes, Forest Rohwer, John H. Paul

**Affiliations:** 1 University of South Florida, College of Marine Science, St. Petersburg, Florida, United States of America; 2 Fish and Wildlife Research Institute, Florida Fish and Wildlife Conservation Commission, St. Petersburg, Florida, United States of America; 3 Department of Biology, San Diego State University, San Diego, California, United States of America; University College Dublin, Ireland

## Abstract

Phage integrase genes often play a role in the establishment of lysogeny in temperate phage by catalyzing the integration of the phage into one of the host's replicons. To investigate temperate phage gene expression, an induced viral metagenome from Tampa Bay was sequenced by 454/Pyrosequencing. The sequencing yielded 294,068 reads with 6.6% identifiable. One hundred-three sequences had significant similarity to integrases by BLASTX analysis (e≤0.001). Four sequences with strongest amino-acid level similarity to integrases were selected and real-time PCR primers and probes were designed. Initial testing with microbial fraction DNA from Tampa Bay revealed 1.9×10^7^, and 1300 gene copies of *Vibrio*-like integrase and *Oceanicola*-like integrase L^−1^ respectively. The other two integrases were not detected. The integrase assay was then tested on microbial fraction RNA extracted from 200 ml of Tampa Bay water sampled biweekly over a 12 month time series. *Vibrio*-like integrase gene expression was detected in three samples, with estimated copy numbers of 2.4-1280 L^−1^. *Clostridium*-like integrase gene expression was detected in 6 samples, with estimated copy numbers of 37 to 265 L^−1^. In all cases, detection of integrase gene expression corresponded to the occurrence of lysogeny as detected by prophage induction. Investigation of the environmental distribution of the two expressed integrases in the Global Ocean Survey Database found the *Vibrio*-like integrase was present in genome equivalents of 3.14% of microbial libraries and all four viral metagenomes. There were two similar genes in the library from British Columbia and one similar gene was detected in both the Gulf of Mexico and Sargasso Sea libraries. In contrast, in the Arctic library eleven similar genes were observed. The *Clostridium*-like integrase was less prevalent, being found in 0.58% of the microbial and none of the viral libraries. These results underscore the value of metagenomic data in discovering signature genes that play important roles in the environment through their expression, as demonstrated by integrases in lysogeny.

## Introduction

“Microbes run the world. It's that simple” [1]. If that statement is true then it should be readily apparent that since viruses often control the microbes, perhaps it could also be said that viruses run the world. One undisputed fact is that viruses are the most abundant biological entities on the planet, with bacteriophages alone at an estimated total abundance of 10^30^ viruses [2].

A great deal of study has gone into the investigation of viruses in aquatic systems since the discovery of their extraordinary abundance only 20 years ago [3]. In recent years there has been a growing appreciation of the diversity, complexity and importance of viral communities to ecosystem function [reviewed in [2], [4]–[7]]. However, most of this investigation has been focused on lytic viruses. In aquatic systems many viruses have the capability of forming a stable symbiosis with their host bacterium, otherwise known as lysogeny. During lysogeny, temperate phage genomes are maintained in their hosts as prophages, principally through integration into one of the cellular replicons or as an autonomous plasmid [8]. Compared to lytic viruses there is a paucity of information concerning lysogeny in the environment. Understanding lysogeny is imperative because of its impacts on host phenotype, community composition and perhaps most importantly, gene transfer processes [reviewed in [9]]. It is becoming clear that viruses are vital repositories and vectors of not only viral genes, but host metabolic genes as well [10]–[12].

Prophages are most commonly detected in natural environments and bacterial isolates by prophage induction. Many prophages maintain the ability to initiate a lytic cycle in response to physical or chemical manipulation, allowing them to exist as free bacteriophages and infect new hosts. This lytic cycle can often be artificially activated by metabolic or DNA damage to the host bacterium, commonly with a toxic substance such as Mitomycin C or ultraviolet radiation (UV). Using this or similar methodology, it has been estimated that roughly half of bacterial isolates contain prophages [13]–[17]. In marine environments the calculated lysogenic fraction of the population can range from 0 to 100%. This frequency varies spatially and temporally with the reproducible trend of higher frequencies of lysogeny in less productive environments and extreme environments [reviewed in [9]]. Seasonal cycles of lysogeny have also been documented in environments as divergent as sub-tropical Tampa Bay and saline Antarctic lakes [18], [19].

Currently, analysis of environmental metagenomic samples is greatly expanding our understanding of the incredibly complex environmental bacterial and viral communities. The analysis of metagenomic sequence data for community structure and the taxonomic distribution of identified genes is an important first step, analogous to answering the question “who's there?” A seminal, early metagenomic work demonstrated that certain genes and metabolic pathways appear to be depth specific. This suggested depth-specific communities and metabolism [20]. This same work also observed large numbers of lytic viral sequences in the photic zone and a prevalence of prophage and transposase sequences at depth. This result provided support for the contention that lysogeny is more prevalent in deep marine environments.

Deciphering ecosystem functioning, or “what are they doing,” by sequence analysis alone is inherently limited. Despite this limitation certain metagenomic studies have attempted to connect metagenomic sequence information to putative ecosystem functions [20], [21]. Some recent metagenomic studies have also begun to examine the functioning of viral communities specifically. Bench *et al*. utilized a viral metagenome library to conclude that cyanophages were very important components of the Chesapeake Bay virioplankton and that the photosynthesis core gene *psbA* may be nearly universal in these cyanophage populations [22]. Another recent metagenomic study went one step further and verified that viral *psbA* genes are not only present, but transcribed [23].

Metagenomic analysis has also begun to demonstrate that differing biogeochemical environments appear to select for specific metabolic functions. A recent large-scale comparative analysis of 42 microbial and 45 viral metagenomes, including the viral metagenome from this study, indicated that the metabolic profiles of the microbial communities were diagnostic of the environment of isolation [10].

This study presents a more fine-scale analysis of the induced viral metagenome representing two important milestones. Firstly, it provides an ecological context by connecting an important physiological parameter to expression of a gene function inferred from metagenomic sequence data. In addition, it is the first metagenomic study focused on lysogeny. We used a viral metagenome prepared from an induced natural microbial population to further our understanding of the functioning of lysogeny in natural systems. For this work our hypothesis was that genes specific to the process of lysogeny would be identifiable in the Tampa Bay viral metagenome and that expression of these genes would be detectable and concurrent with prophage induction in natural samples.

## Results and Discussion

### Metagenome characteristics

The induced viral metagenome from Tampa Bay produced 294,068 reads with an average length of 104 bp for total of 29.1 Mb of sequence information. Initial BLAST (teraBLASTx) analysis of the metagenome demonstrated a percentage of significant (e-value <10^−3^) blast hits to known sequences of 6.6%, which is comparable to other viral metagenomes prepared and analyzed in a similar fashion [24]. Contig spectrum analysis of the community structure of the metagenome indicated that there were approximately 15,400 viral genotypes in the library with the most abundant genotype estimated at 4.43% of the community. For this library the best-fit model was the logarithmic model, consistent with other aquatic viral libraries prepared similarly [25]. The diversity measured by the Shannon-Wiener index was high at 9.13 nats. These values are in the median range of four other viral metagenomes analyzed similarly [24]. Sequences were considered viral if the top blast hit was below the pre-determined e-value cutoff and was to a sequence annotated as viral. Interestingly, this metagenome was markedly higher in the percentage of recognizable viral sequences at 30.5% in comparison to an average of 6.6% for 13 other marine bacterial and viral metagenomes isolated from similar environments [10]. This result suggested that the Mitomycin C treatment of the original bacterial concentrate may have caused a shift in the composition of the viral community, presumably due to induction of prophages. However, this could only be demonstrated conclusively by comparison to a non-induced library from the same sample. Similar high frequencies of viral hits have also been observed in viral metagenomes from dissimilar environments such as hypersaline or coral-associated libraries [10].

**Figure 1:**
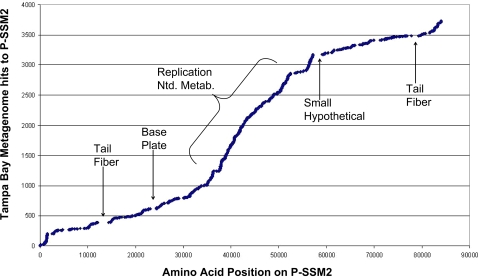
Amino acid coverage of cyanophage P-SSM2 genome by sequences from the Tampa Bay viral metagenome. The slope of the line indicates the genome coverage with high slope indicating high coverage. Arrows identify gaps in coverage and the bracket indicates the area of highest coverage.

The top fifteen most frequent virus identifications using BLASTx analysis from the GenBank nr database of unassembled sequences from the library are listed in Table 1. Seven of the top 15 most frequent hits were to cyanophages, with the cyanophage P-SSM2 being the top contributor. This elevated occurrence of cyanophages has been observed in other metagenomic libraries and underscores the apparent importance of cyanophages in marine ecosystems [12], [20], [22], [24]. A closer examination of the sequences similar to P-SSM2 demonstrated that this phage type appears to be important in this estuarine environment despite the fact that its *Prochlorococcus* host is found in oligotrophic oceanic environments, not estuaries like Tampa Bay. A comparison of the distribution of the amino acid sequences similar to P-SSM2 to the amino acid position on the complete P-SSM2 genome demonstrated high over all coverage of the putative viral functions (Figure 1). A similar analysis was performed on the Chesapeake Bay viral metagenome [22], however that analysis demonstrated a dissimilar pattern of P-SSM2 genome coverage. In contrast to the Chesapeake Bay sample which showed a high level of coverage in the areas of phage structural genes, the Tampa Bay viral metagenome showed an almost opposite pattern, with high coverage in the area of genes involved in replication and nucleotide metabolism and complete gaps in coverage in the area of known phage structural genes. These gaps were the most pronounced in areas of P-SSM2 containing identified tail fiber genes, which are likely involved in host specificity. We hypothesize that this is due to a high number of genetically distinct but functionally similar groups or consortia of cyanophages in Tampa Bay. This hypothesis is supported by the inability to assemble the same sequences to the P-SSM2 reference sequence on the nucleotide level. However, it should be noted that these differences may not be solely due to the fact that the Chesapeake Bay is a dissimilar environment to Tampa Bay. In addition to the contrasting environment, the Chesapeake viral library had a longer average read length. The longer read lengths have been demonstrated to yield higher detection of viral and microbial genes as well as recognition of more distal homologs [26].

**Table 1:** Top 15 Hits for Unassembled Tampa Bay Induced Viral Metagenome.

number of hits	Virus Identification	Target Accession	Category	Virus/Organism Type
2572	Cyanophage P-SSM2, complete genome	AY939844.1	C	T4-like *Prochlorococcus* cyanophage
1184	Cyanophage P-SSM4, complete genome	AY940168.1	C	T4-like *Prochlorococcus* cyanophage
901	Bacteriophage S-PM2, complete genome	AJ630128.1	C	T4-like “photosynthetic” *Synechococcus* cyanophage
524	Roseophage SIO1, complete genome	AF189021.1	-	Lytic bacteriophage of *Roseobacter*
201	Cyanophage P-SSP7, complete genome	AY939843.1	C	T7-like *Prochlorococcus* cyanophage, possibly temperate
174	Bacteriophage M6, complete genome	DQ163916.1	-	*Pseudomonas aeruginosa* bacteriophage, siphovirus NOS
170	Bacteriophage PA11, complete genome	DQ163915.1	-	*Pseudomonas aeruginosa* bacteriophage NOS
93	Bacteriophage Phi JL001, complete genome	AY576273.1	P	temperate phage associated with sponge bacterium
89	Pseudomonas aeruginosa phage PaP3, complete genome	AY078382.2	-	*Pseudomonas aeruginosa* bacteriophage NOS
81	Bacteriophage S-RSM2, incomplete	AJ628768.1	C	Infective *Synechococcus* myovirus, genes ORF1, omp1 gene, psbA gene, psbD gene and tal gene
74	Cyanophage P60, complete genome	AF338467.1	C	Lytic cyanophage
68	Bacteriophage KVP40	AY283928.2	-	Marine broad host-range T4-like vibriophage
65	Chlamydia phage PhiCPG1, complete genome	U41758.1	-	Microvirus (ssDNA bacteriophage)
61	Bordetella phage BPP-1, complete genome	AY029185.2	P	Podovirus, P22-T7 hybrid. Related to temperate *Yersinia* phage PY54
58	Uncultured cyanophage clone BAC9D04	AY456121.1	C	cyanophage sequence from environmental clone NOS. Genes include hypothetical protein, head-tail connector protein, capsid assembly protein, and PSII D1 protein (psbA) genes, complete cds; and unknown genes

Category: C = cyanophage, P = prophage, - = other

The next level of analysis involved a high stringency assembly of the metagenome sequences. A low number of the total sequences were placed in non-trivial assemblies (greater than 2–3 sequences assembling). However, once the sequences in the metagenome were assembled and re-analyzed, a different picture of the viral community emerged (Table 2). In this case, the analysis was based on the contig size and number of sequences that were placed in assemblies. The contigs were grouped according to decreasing size and the top virus hit for each contig is shown in Table 2. For the assembled sequences nine of the top 15 hits were to experimentally verified prophage or temperate phages. This analysis supported our hypothesis that the induced viral metagenome was enriched in prophages. The assembly process is only able to piece together portions of genomes present in relatively high abundances. The high number of prophages in the assembled sequences may have been due to a higher number of same sequences caused by the induction of a large number of phages with identical or similar genomes.

**Table 2:** Top 15 Assembled Contigs with BLASTX hits, Tampa Bay Induced Viral Metagenome.

Contig number	Contig size (bp)	Number of Sequences in Contig	Top BLASTX Hit	Target Accession	e-value	Category	Virus/Organism Type
Contig_13758	3097	248	putative prophage terminase large subunit	NP 455525.1	e-112	P	Prophage [Salmonella enterica subsp. enterica serovar Typhi str. CT18]
Contig_15615	3004	290	orf13 [Haemophilus phage HP1], putative adenine-specific methylase	NP 043482.1	6.00E-13	P	temperate phage HP1 of Haemophilis influenzae
Contig_13453	2301	307	gp6 [Salmonella typhimurium bacteriophage ES18]	YP 224144.1	8.00E-18	P	temperate, generalized transducing phage (dsDNA)
Contig_18645	1744	189	tail protein [Yersinia phage PY54]	NP 892067.1	8.00E-14	P	temperate phage of Yersinia enterolytica
Contig_16978	1631	138	gp9 [Salmonella typhimurium bacteriophage ES18]	YP 224147.1	7.00E-22	P	temperate, generalized transducing phage (dsDNA)
Contig_18692	1596	195	phage protein [Pseudomonas entomophila L48]	YP 609639.1	6.00E-10	P	prophage (phage protein in bacterial genome)
Contig_14529	1429	205	Hypothetical protein CBG24242 [Caenorhabditis briggsae]	CAE56519.1	1.00E-07	-	eukaryote
Contig_14768	1400	98	putative DNA primase [Pelobacter carbinolicus DSM 2380]	YP 357233.1	2.00E-29	-	bacterial whole genome
Contig_14399	1205	51	replication initiation protein [Banana bunchy top virus]	AAG44003.1	6.00E-06	-	lytic plant virus, multicomponent ssDNA
Contig_18753	1128	89	hypothetical protein MED193_12573 [Roseobacter sp. MED193]	ZP 01058432.1	4.00E-07	-	bacterial whole genome
Contig_18853	932	70	viral A-type inclusion protein, putative [Trichomonas vaginalis G3]	XP 001330650	1.00E-04	-	viral protein in Trichomonad (eukaryotic pathogen)
Contig_16209	923	41	hypothetical protein PputGB1DRAFT_4712 [Pseudomonas putida GB-1]	ZP 01715014.1	2.00E-27	P	prophage (Phage-like protein in bacterial genome)
Contig_18743	881	54	gp14 [Salmonella typhimurium bacteriophage ES18]	YP 224152.1	3.00E-05	P	temperate, generalized transducing phage (dsDNA)
Contig_18669	800	31	hypothetical protein ph57 [Staphylococcus phage PH15]	YP 950719.1	3.00E-10	P	temperate phage of Staphylococcus epidermidis
Contig_14647	737	38	conserved hypothetical protein [Cyanophage P-SSM2]	YP 214416.1	8.00E-04	C	lytic T4-like cyanophage

Category: C = cyanophage, P = prophage, - = other

An additional two top hits were to bacterial whole genomes, suggesting viral carriage of host genes or possibly unidentified prophage (Table 2). The first was to *Pelobacter carbinolicus*, a Fe(III) reducing, anaerobic aquatic sediment species. The second was *Roseobacter* sp. MED 193 from the Mediterranean sea. An examination of these genes indicated that they were found in prophage-like elements (i.e., were surrounded by phage genes and/or lysogeny genes; data not shown).

The lytic cyanophage P-SSM2 was still present as the 15^th^ largest contig to be assembled, suggesting this virus was also in very high abundance in this sample. The assembly not only changed the observed distribution of top phage hits, but markedly decreased the e-values obtained because of the increased sequence length.

Phage proteomic tree analysis was also performed and provided a visual representation of this shift in types of phage hits between the raw and unassembled sequences as shown in Figure S1 [27]. However, it is important to note that the analysis of the assembled sequences was performed with a smaller data set, including only the non-trivial assemblies.

### Induced viral metagenome integrase genes

The primary objective for obtaining the induced viral metagenome was to identify viral genes involved in lysogeny that were present in Tampa Bay lysogens. Initial searches of the data attempted to locate cI-type repressors. This was the initial target because repressors are known to be expressed constitutively, and were hypothesized to be more readily detectable in RNA-based gene expression experiments. However, no repressor-like genes were identified.

Another gene known to be involved in the functioning of the lysogenic switch is the enzyme integrase, which catalyzes the integration of the temperate phage into the host genome. Interrogation of the known blast hits revealed 103 (de-replicated) phage integrase-like sequences from the metagenome. Of these sequences, each was examined individually and four were chosen with the lowest e-values (ranging from 10^−4^–10^−9^) as well as a full-length hit of the query sequence to a known phage-like integrase (i.e., greater than 30 amino acids in length). The four integrases were identified as a *Vibrio*-like phage integrase, *Clostridium-*like phage integrase, *Roseovarius/Oceanicola*-like phage integrase and *Alkalimnicola*-like phage integrase. General properties and the sequences of the four identified integrases are listed in Table S1.

The real-time PCR integrase assay was initially tested on a sample of concentrated bacterial fraction DNA from the same environment sampled for the metagenome. The total microbial fraction was used in order to detect putative integrated prophage rather than free phage. Two of the four integrases were detected in this initial screening. The *Vibrio-*like integrase was detected at an abundance of 1.1×10^5^ gene copies L^−1^ of bay water. Based on ambient bacterial abundance at the time of sampling, this gene was present in 0.005% of the ambient population. The *Oceanicola*-like integrase was also detected in this sample, but just at the detection limit. In this case the estimated gene copy number was 1300 copies L^−1^, or an estimated 0.00008% of the ambient population. The other two integrases were not detected in this initial trial. These results indicated that at least some of the genes identified by the metagenome sequencing were not ephemeral and if active, should be detectable as transcripts throughout the annual cycle.

### Seasonal sampling and integrase gene expression in environmental samples

For the gene expression study all parameters were measured over an entire annual cycle at the same sampling site in Tampa Bay. The ambient parameters showed a similar pattern to previous seasonal observations in this environment [19]. This estuary is typified by a spring and fall bloom in primary productivity and *Synechococcus* abundance (Figure S2). The total heterotrophic bacterial populations show a more subtle seasonal oscillation typified by high summer and low winter abundances (Figure S2).

**Figure 2:**
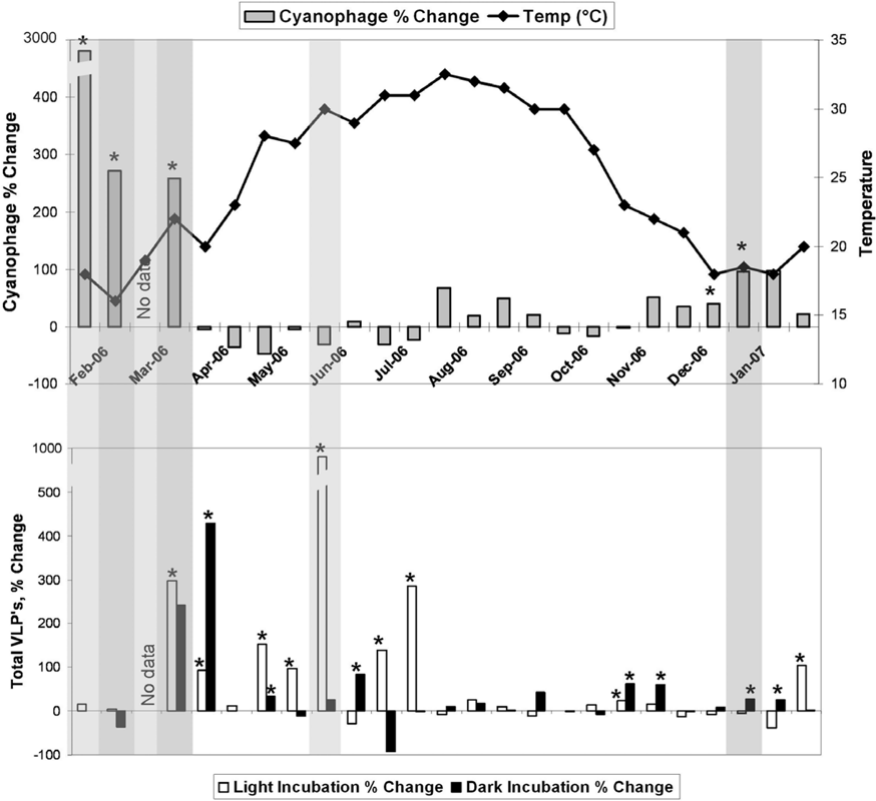
Prophage Induction and Integrase Gene Expression over an annual cycle. The top panel depicts *Synechococcus* cyanophage induction (♦ = temperature, gray columns = cyanophage induction); the bottom panel shows heterotrophic prophage induction (white columns = heterotrophic light incubation, black columns = heterotrophic dark incubation). Light gray shading indicates expression of *Clostridium*-like integrase. Dark gray shading indicates expression of both *Clostridium*-like and *Vibrio*-like integrases. Asterisks denote statistically significant prophage induction. Note the breaks in the y axis in both panels denoted by breaks in the axis line and in the figure column.

Prophage induction experiments were also performed on each sampling date for the total heterotrophic bacterial populations as well as the sub-population of *Synechococcus*. Figure 2 depicts the distribution of prophage induction events over the annual cycle. In the *Synechococcus* experiments positive prophage inductions are clustered in the winter samples, with no induction observed during the summer months. This is consistent with previous observations of seasonal patterns of *Synechococcus* prophage induction in Tampa Bay. For the total bacterial inductions, statistically significant inductions were more frequent throughout the annual cycle and only showed a period of no inductions during the autumn season. Interestingly, in this study the heterotrophic and *Synechococcus* inductions were somewhat temporally separated, with the *Synechococcus* inductions occurring earlier. In the 1999–2000 seasonal study the inductions temporally co-occurred [19], [28].

The most important observation was the distribution of the positive integrase gene expression depicted by the shading in Figure 2. Expression of the *Clostridium*-like integrase was observed on six of the sampling dates and the *Vibrio*-like integrase was detected on three of the sampling dates, overlapping with positive *Clostridium*-like expression. The estimated copy numbers the *Vibrio*-like and *Clostridium*-like integrases were estimated as ranging from 2.4–1280 L^−1^ and 37 to 265 L^−1^, respectively. In all cases a detection of integrase gene expression was present in conjunction with statistically significant prophage induction. On no occasion was integrase expression present without prophage induction. In addition the detections of integrase expression were clustered in the “lysogenic season” with none in the “lytic season.” These findings supported our initial hypothesis.

Multivariate statistical analysis of the distribution of integrase gene expression in comparison to all measured environmental variables indicated that the two parameters most correlated with gene expression were the combined parameters of cyanophage induction and light incubated total prophage induction (ρ = 0.608, p = 0.1). This is statistically significant at the 90% confidence interval, which is a good correlation considering the low number of data points available for comparison. One sampling date was excluded from the analysis due to the loss of the prophage induction experiments, leaving five sample dates with integrase gene expression for statistical analysis.

### Distribution of integrase genes in global environmental samples

Our next investigation was to determine the frequency of both expressed integrases in diverse global environments by investigating their frequency within environmental microbial and viral metagenome libraries. Examination of the integrase gene frequency in microbial samples gives a first estimate of the distribution of similar genes as putative prophage. The frequency of the same genes in viral metagenomes gives a rough estimate of their prevalence in the free virus fraction.

To query environmental microbial samples we estimated the integrase gene frequency in the microbial fraction samples from the global ocean sampling expedition by comparison to the frequency of the single-copy gene *recA* as a proxy for estimation of total genome equivalents [29]. The *recA* gene was identified in 50 of the 56 samples listed on the CAMERA database with an average copy number of 149. The *Vibrio*-like integrase was observed in 39 of the 56 sites with an estimated average of 3.14% of the microbial population carrying a similar gene. In contrast, the *Clostridium*-like integrase was only found in 17 of the 56 sites with an estimated average frequency of 0.58%. There was no clear trend in the type of environment in which the genes were observed.

In four viral metagenomes from widely separated geographical environments [24], genes significantly similar to the *Clostridium*-like integrase were not found. However, the *Vibrio*-like integrase was found in all four viromes with a varying frequency. There were two similar genes in the 416,456 sequence library from British Columbia, containing an estimated 129,000 viral genotypes. One similar gene was detected in both the Gulf of Mexico and Sargasso Sea libraries with 263,908 and 399,343 sequences in each and estimated viral genotype abundances of 15,400 and 5140, respectively. In contrast, in the Arctic library of 188,590 sequences, eleven similar genes were observed with a modeled population estimate of 532 genotypes. This is consistent with the previous finding of high numbers of identifiable prophage genes in this metagenome of Arctic free viruses [24]. Both findings provide support for the contention that lysogeny is prevalent in environments less favorable to bacterial host growth [18], [30]. It is important to note here that the estimated number viral of genotypes presented for each library are theoretical “best guesses” and may vary depending on the assembly criteria used for modeling.

### Concluding Remarks

We have demonstrated the capacity of metagenomic sequence information to provide useful and relevant clues to environmental functions of interest. This study represents an important first connection between viral metagenomic sequence data and an ecologically relevant function within a natural environment. We have demonstrated that viral integrase genes are present and can be stable in the population over periods of time and that expression of these genes was detectable in natural samples. Expression was observed to be temporally variable and most importantly to co-occur with prophage induction. This provided strong experimental support for our initial hypothesis.

Besides demonstrating temporal variability in expression, it appears that similar integrase genes vary in global distribution as well. Integrase-like genes were more frequently observed in microbial samples than free virus samples, which is not surprising since the main function of integrase is to integrate into a host's DNA. In the microbial fraction there was no observable association with a particular environment, but in the virus fraction libraries the gene was much more prevalent in the sample from the Arctic, suggesting greater activity of temperate viruses in colder environments.

Metagenomics may also allow a new perspective on microbes at an ecosystem scale, particularly by highlighting the importance of a specific organism type to overall ecosystem function. This was illustrated in this study by the high functional coverage of the cyanophage P-SSM2. The amino-acid level genome analysis indicated an important functional role being performed in the Tampa Bay estuary by a diverse assemblage of similar phage. Once the raw sequences were assembled, blast hits of the large contigs did not demonstrate the same dominance of cyanophages, leading to the hypothesis that this may have been due to high prevalence of similar but not identical cyanophages in this sample.

## Materials and Methods

To construct the induced viral metagenome a 200 liter sample of seawater was obtained from Tampa Bay on December 13, 2005. The sample was pre-filtered with 5 µm mesh to remove zooplankton and large phytoplankton. The microbial fraction in the sample was then concentrated using a Pro-Flux model M-12 tangential flow filtration device (Millipore, Bedford MA, U.S.A.) using a spiral-wound filter with a 30 kDa cutoff. The volume was reduced to 1160 ml. 1000 ml was treated with 1 µg ml^−1^ Mitomycin C to stimulate prophage induction and 160 ml was left untreated as a control. After a 24 hour incubation in the dark, the number of viral particles was enumerated in both the treatment and control samples using SYBR Gold staining. The treatment concentrate contained 3.5×10^9^ virus-like particles (VLP's) ml^−1^, which was a statistically significant 3-fold increase in virus abundance in comparison to the non-amended control. The treatment sample was centrifuged and 0.2 µm filtered to separate most of the remaining bacterial cells and debris from the viruses. The viruses were then concentrated by polyethylene glycol precipitation and purified by cesium chloride density gradient centrifugation [31]. The viral capsids were disrupted by formamide and the nucleic acids precipitated with ethanol and purified by CTAB extraction [31]. The nucleic acid from the purified viral particles was amplified using the GenomiPhi DNA amplification kit (G.E./Amersham Biosciences, www1.gelifesciences.com). The resulting purified, phi29 amplified DNA from four separate reactions was pooled and sequenced using pyrosequencing technology by 454 Life Sciences [32].

This metagenome was included in a large-scale comparative analysis by Dinsdale et al, which encompassed all of the currently available microbial and viral metagenomes [10]. The sequences from this metagenome are freely available on the SEED platform (http://www.theseed.org), under the accession number 4440102.3. The sequences are also being made accessible from the CAMERA database (www.camera.calit2.net) and from NCBI (www.ncbi.nlm.nih.gov), deposited in the short read archive under the genome project ID number 28619.

The resulting sequences were then compared against the Gen Bank non-redundant database using the BLASTX algorithm. Batch blasts were performed using the Code Quest system (TimeLogic, Carlsbad CA, www.timelogic.com). Sequences were considered “known” if they had a BLASTX similarity with an e-value ≤0.001. Completed blasts were interrogated for the presence of lysogeny related genes using a word search for related gene names or functions. Genes of interest were examined and re-blasted against the non-redundant database individually. Primers and probes for real-time PCR were designed based on the 4 specific selected integrase sequences (Table S1).

Comparison of the viral metagenome hits to P-SSM2 was performed by extracting all sequences with top blast hits to P-SSM2. The stated nucleotide position of each hit was converted to amino-acid position. The reads were then sorted in ascending order and graphed in comparison to the complete nucleotide sequence of P-SSM2. Areas of high coverage are observed as high slope due to the density of sorted points in that area along the genome of P-SSM2. Gaps larger than 500 bp were examined on the fully sequenced genome of P-SSM2 in GenBank for determination of gene content. Attempts to assemble these sequences using P-SSM2 as the reference genome at the nucleotide level were unsuccessful.

To estimate viral diversity and community structure in the library contig spectrum analysis was performed using mathematical rank abundance modeling with the online PHAge Communities from Contig Spectra (PHACCS) tool (http:biome.sdsu.edu/phaccs) [33]. Average contig spectra were calculated using 20 assemblies of 20,000 randomly selected sequences from the library as performed using Circonspect (http://biome.sdsu.edu/circonspect/) with a 98% minimal match percentage and a 35 bp overlap. PHACCS modeling parameters included an average genome size of 50,000 bp. The diversity estimates were determined based on best-fit mathematical modeling, in this case to a logarithmic model.

High-stringency assemblies of the viral metagenome were performed using the SeqMan program in the Lasergene software suite (www.dnastar.com/products/lasergene.php) with a minimum overlap of 30 and minimum match percentage of 95%. There were 132 assembled contigs ≥400 bp which were batch-blasted against the GenBank non-redundant database. Forty-two percent of the consensus contig sequences had a significant match to the non-redundant database. Once the size of the contigs dropped below 400 bp the percentage of positive hits decreased precipitously and most of the contigs were observed to be trivial (i.e. containing only two or three sequences), so these contigs were not considered further. The assemblies were performed in order to more accurately assess the community composition since it has been observed that smaller read lengths are much less likely to be identified [26].

Phage proteomic tree analysis was performed on both the unassembled and assembled sequences from the metagenome [27]. The sequences were compared to the phage and prophage genome database using tBLASTx (E-value cutoff <10^−3^). This database contains sequences from 510 completely sequenced prophage and phage genomes. A comparative phage proteomic tree was constructed using Phage Proteomic Tree version 4 (PPT, http://phage.sdsu.edu/rob/PhageTree/v4). The significant blast hits of both the assembled and unassembled sequences were then mapped on the tree using Bio-Metamapper, an online tool for metagenomic tree analysis (http://scums.sdsu.edu/Mapper).

Samples for the seasonal study of lysogenic gene expression and all other environmental parameters were collected from the same site as the metagenome sample, the St. Petersburg Pier. Two liter samples were collected bi-weekly from January 31, 2006 through January 24, 2007. Parameters measured included temperature, salinity, total monthly precipitation, bacterial and viral direct counts, ambient *Synechococcus*, and infectious cyanophage abundance, total primary productivity, secondary productivity and prophage induction with the total bacteria and *Synechococcus* populations.

Bacterial and viral direct counts were performed using SYBR Gold staining as previously described [34]. *Synechococcus* cells were enumerated using their natural auto fluorescence and infectious cyanophage abundance was estimated using the MPN technique as previously described [35]. Ambient cell abundances and counts for prophage induction experimental samples were measured using the same methods.

Primary productivity was estimated using the ^14^C-HCO_3_ incorporation method [36]. In brief, 100 ml^−1^ samples were placed in duplicate 500ml acid washed sterilized, polycarbonate bottles and approximately 50 µCi of ^14^C-HCO_3_ was added to each sample. Light and dark incubations were prepared for determination of the light dependent rate of carbon fixation. Sample bottles were incubated at ambient water temperature in a flowing ambient water incubator, covered with two layers of neutral density screening. Samples were filtered at T = 0 and T = 2 hours Radioactive counts were corrected for efficiency by use of a ^14^C and were counted in a scintillation counter as previously described [36].

Bacterial productivity was measured by the method of Kirchman et al. [37]. Briefly, ambient water samples from each sample were split in two, a treatment and control. The control flask contained 5% trichloroacetic acid (TCA) to kill the bacteria. 4,5-^3^H-leucine was added to both flasks to a final concentration of 8 nM. Treatment and control flasks were wrapped in aluminum foil and incubated as for primary productivity. Sub-samples were taken at 60 and 120 minutes after T_ = _0 and were filtered as previously described.

Real time PCR (qPCR) was initially performed on samples of the DNA extracted from the microbial fraction of Tampa Bay seawater to determine if the integrases from the viral metagenome were still present. The microbial fraction was collected by first passing the sample through 5 µm mesh to remove macro organisms. The microbes were then collected on a 0.2 µm polycarbonate cartridge filter (Sterivex, Millipore, Bedford MA, U.S.A.). The DNA was extracted using phenol chloroform extraction according to standard protocols [31]. Real-time PCR was performed using PCR primers and FAM™/TAMRA™-labeled probes designed for each specific integrase sequence (Table S1). Reactions were prepared in 50 µl volumes using 1X TaqMan one-step PCR master mix (Applied Biosystems, www.appliedbiosystems.com), 500 nm concentration of each primer and 125 nm concentration of probe. Reactions were analyzed in an Applied Biosystems 7700 Real-Time PCR system and quantified in comparison to a known copy number of artificially synthesized positive control DNA. Two of each type of initial positive amplicon (4 total) from this integrase assay were sequenced and found to be identical to the sequences of interest.

Microbial fraction RNA samples were collected by filtering 200 ml of sample seawater onto 0.45 µm durapore filters (Millipore, www.millipore.com), placed in a 2 ml screw-cap tube with 750 µl of the first step lysis buffer from the RNeasy kit (RLT buffer, Qiagen, Valencia CA) with β-mercaptoethanol added as per manufacturers instructions and frozen at -80°C until sample extraction and processing. RNA extraction was continued utilizing the RNeasy kit protocol (Qiagen, Valencia CA) including on-column DNase digestion according to the manufacturer's instructions. The resulting RNA was purified using the Ambion MegaClear kit to provide the highly purified RNA recommended for amplification (Ambion Inc., Austin TX) and then amplified using the Ambion MessageAmp II RNA amplification kit (Ambion Inc., Austin TX). RNA was quantified and quality assessed using a NanoDrop ND1000 spectrophotometer (Thermo Fisher Scientific, www.nanodrop.com).

Real-time RT-PCR was performed on amplified microbial fraction RNA collected on all samples as for the real-time PCR (as described above) with the addition of reverse transcriptase enzyme and a reverse transcription cycle of 45°C for 30 minutes. Positive control curves were prepared from in-vitro transcribed RNA prepared from the synthetic DNA positive controls.

Standard statistical analyses including paired t-test to determine significance of prophage induction events and multiple correlations of all measured parameters were performed using MiniTab release 13.1. Multivariate statistical analyses were performed using Primer v.5.2.9 software (Primer-E Ltd., Plymouth Marine Laboratory, U.K. www.primer-e.com). This method is based on multidimensional scaling of complex data sets in order to determine relationships with no assumptions of normal distribution. Initially, separate similarity matrices were constructed of all measured environmental variables, including prophage inductions in comparison to the expression pattern of both integrase genes. The sample obtained on 2/28/06 was excluded from the analysis because of loss of the prophage induction experiments. These similarity matrices were compared using the RELATE test (sample statistic ρ) to determine if the two matrices were significantly related. The matrix of gene expression was then transformed to presence/absence because of the uncertainty involved in the copy number calculation due to the amplification step. This was then compared to the distribution of all measured environmental parameters using the BIO-ENV test to determine which variables best explained the pattern of integrase gene expression.

The number of genome equivalents in each microbial sample included in the Global Ocean Sampling series in the CAMERA database was determined by BLASTX searching each sample using the *Escherichia coli recA* gene as a query. For all searches the e-value cutoff for determining a significant hit was 10^−3^. This gene has been used to estimate the number of bacterial genotypes in metagenomic libraries because it is a well-conserved, known single-copy, core metabolic gene [21]. The sequences of both detected integrases were then used as a query in the same fashion and the observed frequencies of both used to calculate an environmental frequency distribution of the integrase genes. For estimation of the frequency of gene occurrence in the four viral metagenomes the number of gene hits was compared to the estimated genotype abundance previously calculated using contig spectrum analysis [24]. This is a minimum estimate of prevalence since not all of those genotypes would be adequately sampled through metagenome sequencing.

## Supporting Information

Table S1 Integrase sequences from Tampa Bay Metagenome.
(0.03 MB DOC)

Figure S1 Phage Proteomic Tree Analysis of Unassembled and Assembled Sequences From Induced Viral Metagenome. The left side of the figure (y-axis) is the tree constructed based on the phage and prophage database. The x-axis represents the abundance (number of blast hits) of each phage type normalized to the must abundant type, which is set at 1. The left column (blue) is the relative distribution of phage hits in the raw sequences. The right column (red) indicates the relative distribution of phage hits in the assembled sequences. The most abundant phage identifications in each column are labeled in the figure.
(5.90 MB TIF)

Figure S2 Ambient parameters in Tampa Bay throughout the annual cycle. The top panel depicts primary productivity (green) and bacterial productivity (blue). The center panel depicts total viral abundance (black) and total bacterial abundance (brown). The bottom panel depicts the ambient Synechococcus abundance (red) and infectious cyanophage abundance (gray). Error bars indicate the standard deviation.
(11.10 MB TIF)
