# Supplementary material for: Metagenomic Analysis of Lysogeny in Tampa Bay: Implications for Prophage Gene Expression
Source: PLoS One. 2008 Sep 23;3(9):e3263. doi: 10.1371/journal.pone.0003263 (PMC2533394; doi:10.1371/journal.pone.0003263)
Supplement: Table S1 — Integrase sequences from Tampa Bay Metagenome. (0.03 MB DOC) [file pone.0003263.s001.doc]

| **Table S1: Phage Integrases from Tampa Bay Induced Viral Metagenome:** | | | | |  |
| --- | --- | --- | --- | --- | --- |
| **Name/I.D.** | **Gene** | **e-value** | **integrase type** | **sequence** | **Probe sequence** |
| *Vibrio cholera* phage integrase | *xerC* | 7.00E-06 | tyrosine type (P4-like) | CTGGTACGCGCTGGGAAAGAAATTGACTTCAAAAACAATCTATGGCGAATTCCGGGCGAGCGGATGAAGAAAAAGAAGCCTCACACTGTACCACTCAGCACCCAAACTTTAGA | CAAAAACAATCTATGGCGAAT |
| *Clostridium cellulolyticum* phage integrase | *xerD* | 6.00E-11 | tyrosine type | TATTCTACACATAAGTTGAGACACACCGCTGCTACATTAATGTATCAATATGGTAATGTCGATATTCGCGCTTTACAGGAAATTTTAGGACATGAAAGT | CACACCGCTGCTACATTAA |
| *Roseovarius /Oceanicola* phage integrase | *xerD* | 9.00E-09 | tyrosine type | TTGTTGATTTACATTTCCATGACTTAAGGCATGAAGCTATATCAAGGTTCTTTGAAAAAGGATTGTCTATACCAGAAGTAAGTCTTATTTCGGGTCATAAGGATGTTAGA | CAAGGTTCTTTGAAAAAG |
| *Alkalimnicola ehrlichei* integrase | *vanD* | 2.00E-04 | tyrosine type (P4/P22-like) | AGTGCATCAAGCTCCGGGTCAAGGATATTGATCTTGATCACCGCACCCTCACCATTCACGGCGGCAAAGGCGACAAGGACCGCATCGTGAAACTCCCCG | TCACCGCACCCTCACCATTCA |
| *Forward primers are indicated with blue text, the reverse primers are indicated with red text.* | | | | |  |
